# Meeting of the Western Dental Society

**Published:** 1859-01

**Authors:** 


### PROCEEDINGS OF SOCIETIES.

### ARTICLE XII.

### *Meeting of the Western Dental Society.*

THE above named Society met in this city on Tuesday morning, the 20th of July, at 11 o'clock, in Lomelino's Hall. The President, Dr. Isaiah Forbes, of St. Louis, in the chair. The seciety was called to order, and a portion of the session was occupied in reading and correcting minutes of previous meeting, and examination of candidates for membership; after which, the society proceeded to the election of officers for the ensuing year.

Dr. W. W. Allport, of Chicago, Ill., was elected President; Dr. I. B. Branch, of Galena, Ill., 1st Vice-President; Dr. W. H. Anderson, Hannibal, Mo., 2d Vice-President; Dr. I. Forbes, St. Louis, Mo., Recording Secretary; Dr. H. T. Peebles, St. Louis, Mo., Correspoding Secretary; Dr. A. Blake, Treasurer; Drs. Spalding, McKellops, Forbes, Priest and Hubbard, Ex. Committee.

Meeting adjourned until 2 o'clock, P. M.

Two o'clock, P. M.—Called to order by President Forbes.

The President, elect, after being conducted to the chair, said:

GENTLEMEN:—For the honor done me in selecting me as your presiding officer, I return you my sincere thanks. It is now three years since the "Western Dental Society," was first organized, and this is its fourth meeting. Whatever of good it may have accomplished towards the advancement of dental science, and the making of our profession one of dignity, usefulness and honor, certain it is that not the least among its useful results is the better acquaintance and consequent good fellowship of its members with each other.

You will agree with me when I say that this society has drawn together the better portion of our profession in the West—men who, by effort and example, have contributed much to the advancement of dental knowledge, and in whom a spirit of emulation and industry has been incited, which promises much for the future. A spirit of rivalry alike laudable and honorable, which does equal credit to the head and heart.

At the time of the formation of this society, the expediency of the movement was doubted by many, and its promised usefulness was questioned—but the results that have followed, and which we witness to-day in the numbers present, and the interest manifested, must, I am sure, set at rest the question of expediency in this matter. The "Western Dental Society," has to-day, a *living* existence, which, I trust, will not soon die out.

I again thank you, gentlemen, for the honor you have seen fit to confer upon me; and while I shall endeavor to discharge the duties imposed upon me to the best of my ability, I trust that I shall be assisted and sustained by every member present; and that business will be brought before the society in a strict parliamentary form, so as to expedite, as far as possible, the business operations of the meeting, and, at the same time, to relieve your presiding officer of much of the burden resting upon him.

After the President's remarks, and the transaction of some miscellaneous business, the regular subjects for discussion were taken up—the first in order being *difficult dentition.*

It was ably discussed by Drs. Spalding, Allport, McKellops, Blake, Barron, Branch, Sturgiss, Anderson, Hubbard, Forbes and others, developing much of interest to the profession. The discussion was protracted and ably sustained, members speaking more directly to the point than usual on such occasions.

The cause of difficult dentition seemed generally acknowledged as constitutional, and generally hereditary. Great reliance was placed upon the use of lime in various forms as bone phosphate, lime water and free use of the gum lancet, as remedial agents, with some dissent.

Next in order came a discussion of *causes and treatment of irregularities of the teeth.*

The discussion was participated in by all the members present, and was interesting and instructive. Cases were reported by Drs. McKellops, Peebles and Allport, demonstrating clearly that almost any deformity of the face, at all dependent on the teeth or jaws, is under the control of the judicious dentist.

At the close of this discussion, the society adjourned, to meet same place at 10 o'clock, A. M., on Wednesday.

---

Second day—Wednesday, 10 A. M.—Convention met pursuant to adjournment—President in the chair.

*Inflamed dentine*, and its treatment, was ably, yet briefly, discussed—each member stating his particular method and prescriptions; a majority agreeing in the occasional careful use of arsenicum, combined with other substances in various proportions, such as creosote, morphine, tannin, alum, &c. One gentleman remarked that recently he treated almost exclusively by applying cold, to produce temporary insensibility. All took part, and the discussion was lively and interesting.

Next in order came the *treatment of exposed nerves.*

Various methods were brought forward as to the practice of the different members; all evincing thought and careful experiment. Cases of capping the nerve were reported, of twenty and twelve years' standing, by Dr. Forbes and Dr. Branch; and this practice recommended when the pulp, or nerve, is in a normal condition. Some sometimes, and others always, kill and take out the nerve, fill the cavity and crown. Of this latter class, Dr. Allport reported 184 cases thus treated, of which he had seen 153—of which eight had small abscesses, and four had been extracted—showing conclusively that it is not always best to extract an aching tooth.

Afternoon Session.—First subject for discussion—*Best manner of inserting partial sets with clasps.*

The general opinion seemed to be that this method should be used as little as consistent with success—all admitting its deleterious and destructive tendency upon the clasped teeth. That, when used, the bearings of clasps should be as few and small upon the teeth as is consistent with proper security to the piece. Various methods were proposed to effect this object, each possessing merit, and evincing thought and effort in the right direction. After which, the relative merits and demerits of different modes of inserting artificial dentures, were discussed.

The prevailing preferences seemed to be for single gum teeth, on good plate, and continuous gum teeth. Other methods were favorably spoken of, including block work, vulcanized rubber and cheoplasty—some making *fits* with the latter; others *giving it fits.*

A committee on local anæsthesia, as used by Dr. I. B. Branch, appointed at the last annual meeting, reported, through their chairman, Dr. Allport, as follows:

"The committee to whom was referred the subject of local anæsthesia, by Dr. I. B. Branch's instruments, beg leave to report, that local anæsthesia, as produced by congelation, although attended with some inconvenience in its preparation and application, can be employed with gratifying results in a large number of cases. They regard it as particularly useful in cases where the administration of chloroform would not be admissible."

W. A. Allport, *Chairman.*

Fifteen minutes were now set apart to present specimens of improvements in dental art, during which many things useful, practical and ornamental, were exhibited. It should have been said previously that Dr. Peebles, of St. Louis, read appropriate and instructive papers on the various topics discussed, which were filed with the proceedings.

Dr. McKellops offered the following preamble and resolution:

*Whereas,* Owing to the great inconvenience of the officers and soldiers in procuring competent dentists, when necessarily required, and knowing the difficulty in whieh they are placed, being stationed at distant posts, where it is absolutely impracticable for a regular practitioner of dentistry to visit them; therefore,

*Resolved,* That this society appoint a committee of five, for the purpose of memorialising Congress on the necessity of appointing dentists to be attached to the regular army, and that we recommend the same to the consideration of the American Dental Convention, and ask their co-operation with us.

The resolution was adopted and the following committee appointed: Drs. McKellops, Spalding, Forbes, Branch and Lewis. President Allport added by vote.

After some further business incidental to the workings of organized bodies, society adjourned to meet at Milwaukie, Wisconsin, on first Tuesday in July, 1859. The session, throughout, was characterized by harmony and good feeling. The President, Dr. W. W. Allport, of Chicago, discharged his duties with intelligent promptitude and gentlemanly urbanity, thus doing honor to himself and the society.

Among the strangers present was Dr. Andrews, of Mississippi, who, by his gentlemanly intelligence in speaking on the various questions, did much to add interest to the meeting. From what we have seen and heard at this meeting, we are convinced that the dental profession includes a large number of men ardently devoted to the advancement of science, and the alleviation of human suffering, and who will compare favorably with any other profession extant. From the amount of theory given, and facts illustrated, we wonder that any in the profession, who desire to excel, can consent to absent themselves from such meetings.

After the arduous labors of the day and evening were ended, and the very interesting and animated discussions of the final session had terminated, the members present, and a few invited guests, were conducted to an upper room, *tastefully* arranged, and amply supplied with creature comforts. The dentists of Quincy nobly upheld the reputation of our city; though few in numbers, they are a host in hospitality—may their shadows never be less.

After the several courses, and the removal of the cloth, the wine came on in ample supply. Toast after toast was given and responded to, by each gentleman as he was called out, in chaste, pointed and appropos impromptu speeches, all of which we would like to report, if we had room in our columns. During the evening the following sentiment was given by our venerable ex-President, I. Forbes:

"May the spirit which animated the early promoters of dental literature—the parents of a liberal, free American dental education, and unselfish interchange of thought—find in the young men of the profession, temples fit to dwell in."

